# Artificial intelligence-based multimodal multitask analysis of thyroid ultrasound image features predicts thyroid cancer: a multicenter study

**DOI:** 10.1093/jncics/pkag037

**Published:** 2026-04-09

**Authors:** Yu Gui, Xuerui Zhang, Yun He, Tingting Li, Lan Hu, Longle Liu, Dan Peng, Juan Yuan, Xiaoyu Xiong, Wenhan Li, Xin Wu, Shichao Li, Haiyan Fan, Tijie Peng, Xi Yang, Xiang Cui, Ying Yang, Lingjuan Zeng, Dajiang Song, Feng Liu, Jianhui Li, Peng Wang, Zhongquan Huang, Li Chen

**Affiliations:** Department of Breast and Thyroid Surgery, Southwest Hospital, Army Medical University, Chongqing, China; College of Mathematics and Statistics, Chongqing University, Chongqing, China; Department of Ultrasound, Southwest Hospital, Army Medical University, Chongqing, China; Department of Breast and Thyroid Surgery, Southwest Hospital, Army Medical University, Chongqing, China; Department of General Surgery, The People’s Hospital of Dazu, Chongqing, China; College of Mechanical and Transportation Engineering, Chongqing University, Chongqing, China; College of Mathematics and Statistics, Chongqing University, Chongqing, China; Department of Breast and Thyroid Surgery, Southwest Hospital, Army Medical University, Chongqing, China; Department of Breast and Thyroid Surgery, Southwest Hospital, Army Medical University, Chongqing, China; Department of Oncology Surgery, Shaanxi Provincial People’s Hospital, Xian, China; Department of Breast and Thyroid Surgery, Southwest Hospital, Army Medical University, Chongqing, China; Department of Breast and Thyroid Surgery, Southwest Hospital, Army Medical University, Chongqing, China; Department of Breast and Thyroid Surgery, Southwest Hospital, Army Medical University, Chongqing, China; Department of Breast and Thyroid Surgery, Southwest Hospital, Army Medical University, Chongqing, China; Department of Breast and Thyroid Surgery, Southwest Hospital, Army Medical University, Chongqing, China; Department of Breast and Thyroid Surgery, Southwest Hospital, Army Medical University, Chongqing, China; Department of Breast and Thyroid Surgery, Southwest Hospital, Army Medical University, Chongqing, China; Department of Breast and Thyroid Surgery, Southwest Hospital, Army Medical University, Chongqing, China; Department of Oncology Plastic Surgery, Hunan Province Cancer Hospital and The Affiliated Cancer Hospital of Xiangya School of Medicine, Central South University, Changsha, Hunan, China; Breast Surgery Department, Fuyang Cancer Hospital, Fuyang, China; Department of Oncology Surgery, Shaanxi Provincial People’s Hospital, Xian, China; Centre for Medical Big Data and Artificial Intelligence, Southwest Hospital, Army Medical University, Chongqing, China; College of Mechanical and Transportation Engineering, Chongqing University, Chongqing, China; Department of Breast and Thyroid Surgery, Southwest Hospital, Army Medical University, Chongqing, China

## Abstract

**Background:**

Thyroid nodule ultrasound (US) images and their features are of great importance in thyroid nodule diagnosis and can be helpful for radiologists’ clinical decision making. The aim was to evaluate whether an artificial intelligence (AI)-assisted system can accurately characterize thyroid nodule US features and assist radiologists in diagnosing thyroid cancer.

**Methods:**

The AI-assisted system (MDT-TC) was trained and internally validated on B-mode US images from 7204 lesions in 6884 patients in Southwest Hospital. The model performance was validated using 3 independent external validation cohorts.

**Results:**

Echogenicity (ECH) and shape (SHA) are features of high importance for model recognition, and these features lead to excellent model performance. The model achieved up to 87.56% accuracy in determining ECH attributes and 69.21% in identifying shape categories. The area under the receiver operating characteristic curve (AUC) of the internal validation cohort and 3 independent external validation cohorts for MDT-TC were 0.951, 0.837, 0.816, and 0.871, respectively. The sensitivity values were 98.7%, 91.2%, 90.3%, and 85.6%, respectively. The AUC for the accurate diagnosis of radiologists with MDT-TC assistance was significantly higher than that of radiologists without MDT-TC assistance (*P *< .001). In addition, the AUC for the accurate diagnosis of junior doctors with MDT-TC assistance was significantly higher than that for those without (*P *< .01).

**Conclusion:**

MDT-TC incorporates radiomic features extracted from thyroid lesion US images and can significantly improve the diagnostic performance of radiologists. This result was particularly strong for junior doctors. Therefore, our data support the idea that MDT-TC can help to identify patients with thyroid cancer and could greatly benefit clinical practice.

## Introduction

Thyroid cancer (TC) is the most common type of head and neck malignancies.^1^ In 2022, it is estimated that 124 907 men and 341 211 women in China will be newly diagnosed with TC, accounting for more than two-fifths of all TC cases worldwide.^2^^,^^3^ Although the incidence of TC has increased significantly over the past decade in China,^4^^,^^5^ TC had the highest 5-year relative survival rate among all cancers, with survival rates of 90.9% in males and 93.5% in females.^6^^,^^7^ Ultrasound (US) imaging and fine-needle aspiration (FNA) biopsy are the primary methods used to assess the risk of thyroid nodules.^8^ US is widely regarded as the primary imaging modality for thyroid assessment because of its accessibility, noninvasive nature, lack of ionizing radiation, and superior diagnostic accuracy for thyroid nodules compared with contrast-enhanced computed tomography.^9^ Thyroid nodule detection relies on the identification of specific benign or suspicious sonographic features, including composition, echogenicity, shape, margins, echogenic foci, and size.^10^ Features such as irregular shape, taller-than-wide orientation, irregular margins, marked hypoechogenicity, microcalcifications, and solid composition are associated with an increased risk of malignancy. However, the significant overlap in US characteristics between benign and malignant nodules limits their diagnostic utility in guiding clinical decision making for thyroid nodules.^11-13^ Moreover, the inherent subjectivity of US interpretation introduces operator and evaluator dependency, resulting in moderate to substantial inter- and intra-observer variability in image acquisition and interpretation. This variability frequently leads to unnecessary FNA biopsies to definitively exclude malignancy.^14^ Previous reports have shown that even with FNA, more than 15% of the thyroid nodules are still difficult to accurately risk-assess.^15^^,^^16^ Therefore, to reduce the rate of FNA, there is an urgent need to develop more refined and accurate risk assessment tools for thyroid nodules based on US images.

Artificial intelligence (AI), a computing technology capable of mimicking or surpassing human intelligence,^17^ is a promising tool for thyroid US research. Accurate identification and analysis of key features in thyroid US images are crucial for precise nodule diagnosis and informed clinical decision making. Current research on AI-assisted thyroid US predominantly focuses on nodule segmentation and classification tasks, often overlooking the potential intrinsic relationships between nodule features and classification outcomes.^18-20^ Both the segmentation and classification of thyroid nodules on US images are fundamental yet challenging tasks in the computer-aided diagnosis of thyroid disease. Given their intrinsic relationships and shared features, multitask learning offers a promising approach to simultaneously address these challenges.

Importantly, the Multi-gate Mixture-of-Experts (MMoE) network model offers a flexible parameter-sharing mechanism for multitask learning. This network model partitions shared network layers into multiple expert networks and learns their contributions to different tasks.^21^ When network nodes are similar, the MMoE model demonstrates superior accuracy and stability compared with single-task models.^22^ In AI applications, feature selection is critical for enhancing model accuracy. Therefore, the MMoE model simultaneously learns diverse radiomic features extracted from US images, including the intensity, shape, and texture feature sets. The model also incorporates an adaptive glandular region feature enhancement module to leverage prior knowledge of the thyroid, guiding the feature enhancement network to precisely classify the thyroid nodules.^23^

In this retrospective study, we constructed an MMoE network to improve the accuracy and efficiency of thyroid nodule predictions. The model incorporates radiomic features extracted from US images as prior knowledge to enhance the diagnostic performance.

## Methods

### Study design and participants

**Figure 1.**
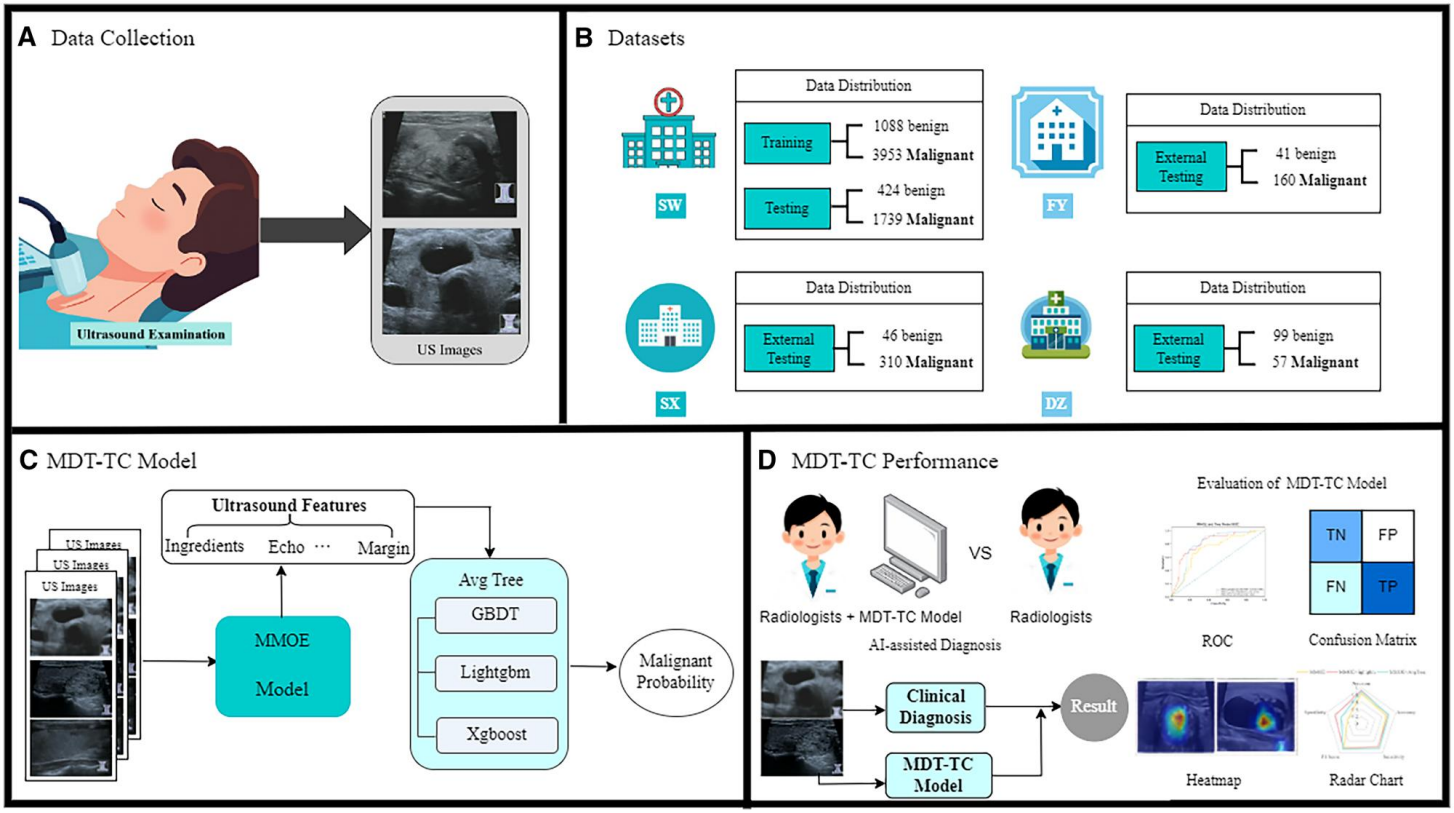
Data and strategy. **A)** Conventional ultrasound (US)-based diagnosis, in which we identified patients with definite benign or malignant pathological findings; **B)** summary of training and validation datasets in which the entire dataset consisted of surgery-confirmed malignant/benign samples collected from 4 medical centers (Southwest [SW], Shanxi [SX], Fuyang [FY], and Dazu [DZ]); **C)** MDT-TC model; and **D)** MDT-TC performance.

Patients with definite benign or malignant pathological findings were retrospectively recruited from Southwest (SW) Hospital between January 2011 and January 2024. We initially trained the proposed model and evaluated its performance on the SW dataset by splitting the data into training and validation datasets. Three external validation cohorts were consecutively enrolled from Shanxi (SX) Provincial People’s Hospital, Dazu (DZ) People’s Hospital, and Fuyang (FY) People’s Hospital. The eligibility criteria were as follows: (1) age > 18 years, (2) definite benign or malignant pathologic findings following thyroid surgery, and (3) acquisition of thyroid US data before thyroid surgery. The exclusion criteria were as follows: (1) inadequate US image quality or lack of US data and (2) metastatic disease or other malignancy. Clinical data were collected and reviewed from the electronic medical record system of each institution (Figure 1).

In the SW database, we had a total of 7716 patients with definite benign or malignant pathologic findings after thyroid surgery, 514 (6.67%) patients in whom the focal area of the US image was covered by the color marker causing unrecognizable features, 33 (0.43%) patients with no US images, and 283 (3.67%) patients in whom US feature labels were missing (Figure S1). The MDT-TC model was trained and internally validated using the SW database, which consisted of B-mode and color Doppler US images of 7204 lesions from 6884 patients with benign or malignant US findings (Figure 1, Table 1). We selected 1 to 2 lesions from each patient as model inputs, and each lesion included 2 images. We collected 2 to 4 (mean, 2.3) images in each patient’s US examination. Because our model yielded the best diagnostic performance according to its AUC when using B-mode thyroid lesion US images compared with using color Doppler images or combining both, we excluded the color Doppler images accordingly. Additionally, based on the American College of Radiology (ACR) Thyroid Imaging Reporting and Data System (TI-RADS),^24^ we extracted US features in each thyroid nodule across the following 5 categories: composition, echogenicity, shape, margin, and echogenic foci. Features in the first 4 categories had a single score derived from mutually exclusive choices, whereas more than one feature may be present in the echogenic foci category. Therefore, our model incorporates 8 US features: composition (COM), echogenicity (ECH), shape (SHA), margin (MAR), echogenic foci or large comet-tail artifact (ELCA), macrocalcification (MAC), peripheral (rim) calcifications (PCL), and punctate echogenic foci (PEF) (Figure S2).

**Table 1.** Detailed patient characteristics and statistics.

Characteristics	Southwest	Fuyang	Dazu	Shanxi
Patients	6884	150	152	349
Lesions	7204	201	156	356
Sex				
Male	1621	35	29	106
Female	5263	115	123	243
Age of patients, years				
Mean	43	52	49	49
Range	18-64	18-84	19-96	21-87
BMI, kg/m^2^			Not given	
<18.5	206	3		10
18.5-24	3618	57		132
24-30	2583	79		182
>30	207	11		25
Smoking				
Yes	1175	19	6	49
No	5709	131	146	300
Drinking				
Yes	1419	32	3	44
No	5465	118	149	305
Hyperthyroidism				
Yes	218	1	2	4
No	6666	149	150	345
Hypothyroidism				
Yes	166	1	3	4
No	6718	149	149	345
Hashimoto thyroiditis				
Yes	2029	0	29	96
No	4885	150	123	253
Pathological type				
Benign	1512	40	99	46
Papillary thyroid cancer	4904	110	57	310
Follicular thyroid cancer	552			
Medullary thyroid cancer	58			
Anaplastic thyroid cancer	178			
BRAF mutation			Not given	
Yes	1819	Not given		Not given
No	5385			

Abbreviation: BMI= Body Mass Index

We divided the SW database into a training test set and an internal test set at a ratio of 7:3. The training test set and the internal test set contained 4973 patients (5041 lesion images) and 1911 patients (2163 lesion images), respectively, and the external test set included 349 patients (356 lesion images) in the Shanxi test set, 150 patients (201 lesion images) in the Fuyang test set, and 152 patients (156 lesion images) in the Dazu test set (Figure S1).

### Ethics statement

This study was approved by the ethics committee of the First Affiliated Hospital of the Army Medical University (No. (B) KY2023092). The requirement for informed consent was waived.

### Model structure

**Figure 2.**
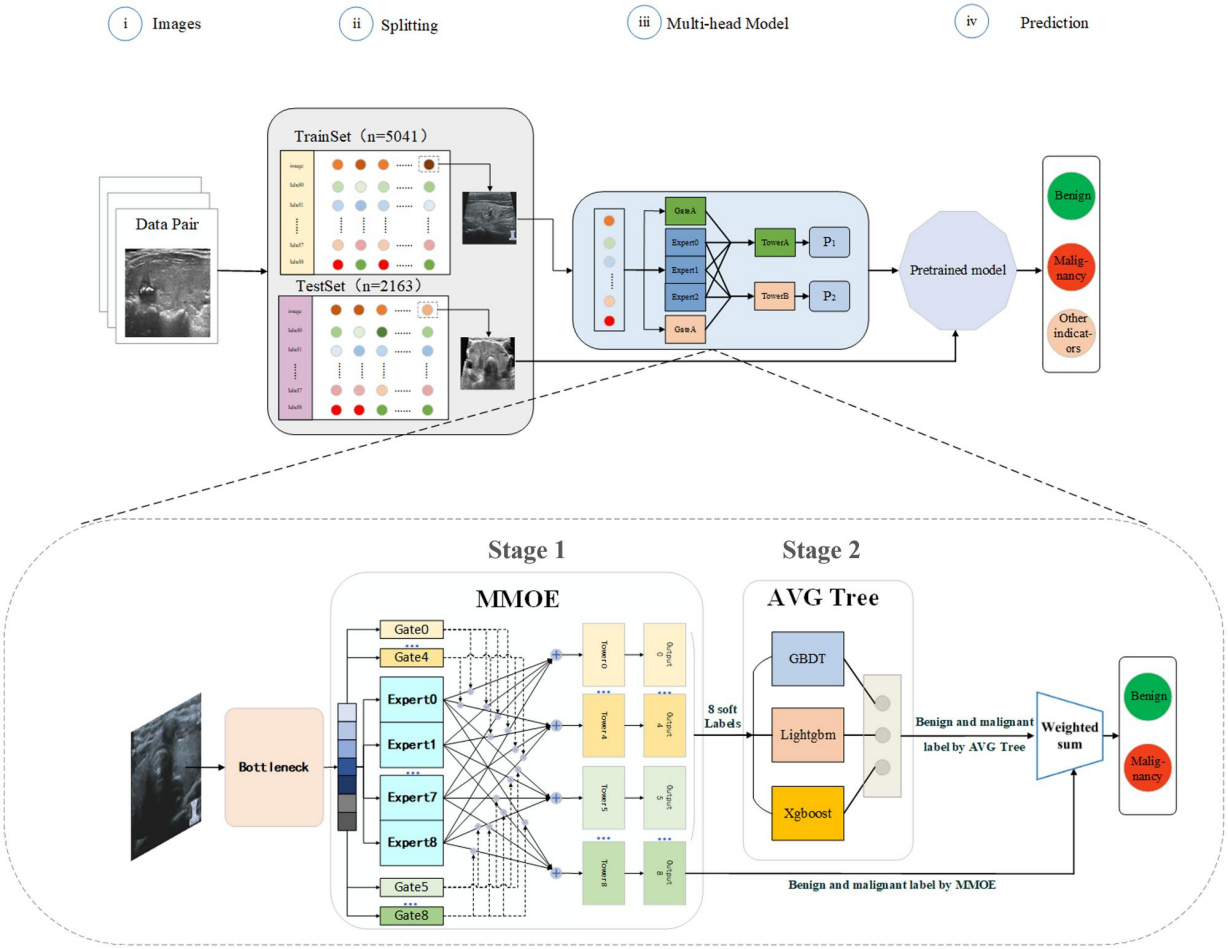
Details of the MDT-CT system. The 2-stage model (MDT-TC) is based on a multitask learning model using the Multi-gate Mixture-of-Experts (MMOE) framework for the first stage and decision tree models for the second stage.

We built a 2-stage model to achieve intelligent diagnosis of thyroid nodules, among which the first stage involves developing a multitask learning-based model using the MMOE framework, and the second stage includes the decision tree model (Figure 2). The detailed model specifications can be found in Supplementary material.

### Model aided diagnosis

When the MDT-TC system assists in diagnosis, radiologists can not only obtain benign and malignant probability scores for thyroid nodules but also obtain clinical US image features based on MMOE to aid in clinical diagnosis. In our study, 6 radiologists were unaware of the confirmed pathological status of the thyroid nodules, and all the study objectives were explained before review. Each radiologist completed the review process independently on an online platform. Additionally, the radiologists’ diagnoses were compared with MDT-TC diagnoses. If the results were inconsistent, the radiologist could choose to progress with his or her original diagnosis or use the MDT-TC system for diagnosis. The final diagnosis was made by a radiologist using the MDT-TC system.

### Statistical analysis

The models were evaluated based on the following metrics from the 5-fold cross validation: area under the receiver operating characteristic curve (AUC), F-measure to receiver operating characteristic (ROC), and values for kappa accuracy, sensitivity, and specificity. To determine whether there was a significant difference in the consistency of the classification results, we assessed intergroup differences between models using McNemar’s test. Using standard reliability plots and Bootstrap bias-corrected plots, the calibration performance of the MDT-TC model was comprehensively evaluated. The detailed calculation method can be found in Supplementary Materials p1-p2. All statistical analyses and metrics were performed and computed using Scikit-learn (version 1.6.1) and SciPy (version 1.13.1) in Python (version 3.9).

## Results

### Baseline characteristics

The MDT-TC system was trained, validated, and internally tested in 6884 patients (median age = 43 [range = 18-64] years; 23.5% men). The external test sets included 349 patients (median age = 49 [range = 21-87] years; 30.3% men) from the Shanxi test set, 150 patients (median age = 52 [range = 18-84] years, 23.3% men) from the Fuyang test set, and 152 patients (median age = 49 [range = 19-96] years, 19.1% men) from the Dazu test set (Table 1). An overview of all the US equipment used by the centers is provided in Table S1.

### Model performance and interpretability

The performance of the proposed system was evaluated by examining its AUC. We found that, compared with the traditional single-task image classification model, the MMOE architecture with multitask learning achieved an internal test AUC of 0.903 (95% CI = 0.890 to 0.916), which was considered the best performing model (Figure S3 and Table S2). In the MDT-TC system, MMOE yielded the best diagnostic performance according to its AUC when using B-mode thyroid lesion US images (Figure S4 and Table S3).

**Figure 3.**
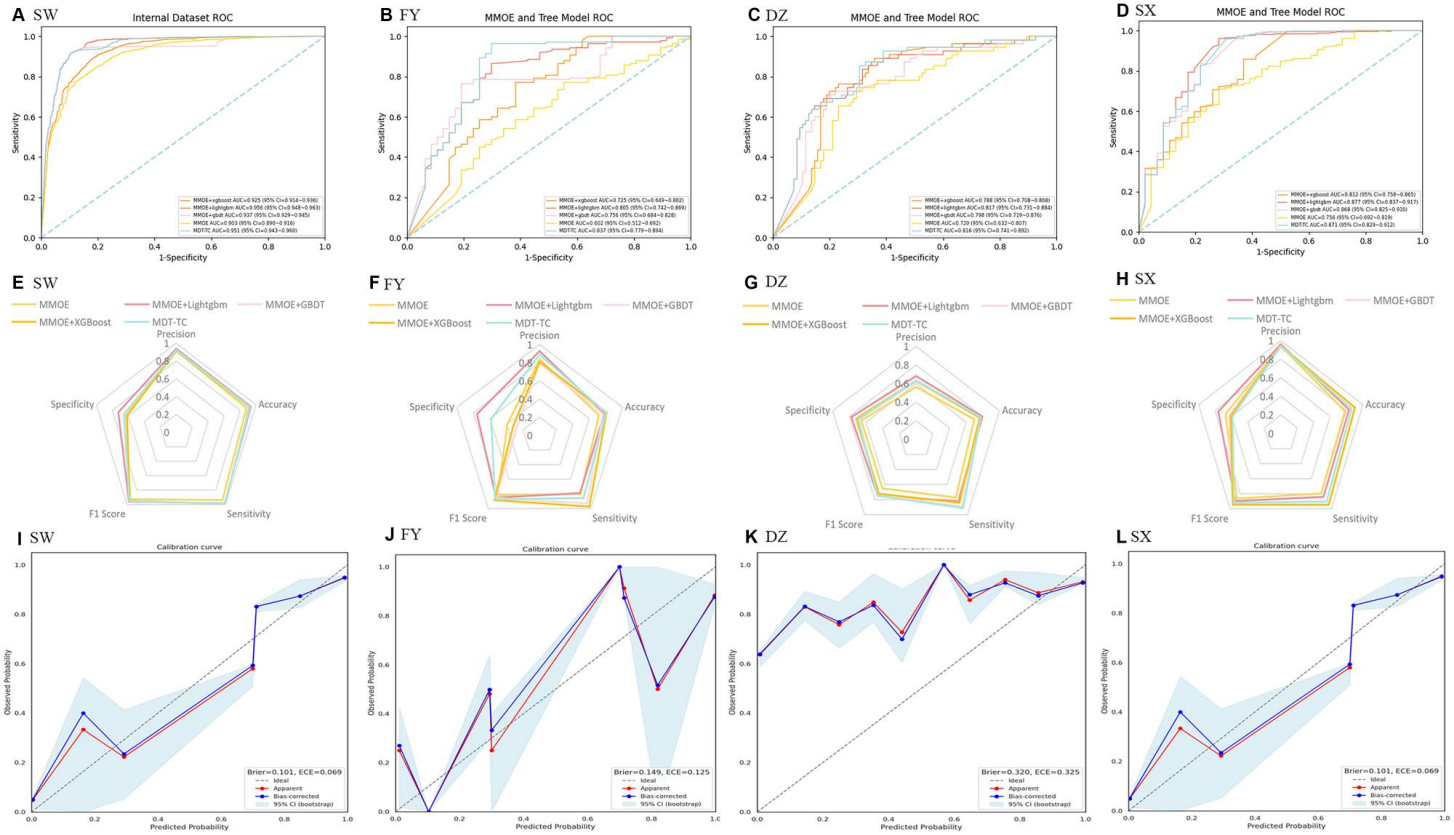
Performance of MDT-TC and radiologists in discrimination of malignant from benign thyroid lesions. Receiver operating characteristic (ROC) curves for the binary classification of thyroid lesions. The area under the receiver operating characteristic curve (AUC) scores are reported together with the 95% confidence interval (CI) on the internal validation set and 3 external validation sets **(A-D)**. Radar maps of the 5 diagnostic indexes (accuracy, sensitivity, specificity, precision, and F1 score) in the internal test set and 3 external validation sets **(E-H)**. Calibration curves on the internal test set and 3 external validation sets (**I-L**).

In the second stage, we combined MMOE with 3 decision tree models (GBDT, XGBoost, and LightGBM) to evaluate the diagnostic performance. The MMOE integrating LightGBM or the Average model achieved an optimal tradeoff between performance and overhead (Figure 3A and Table 2). The AUC for the proposed MDT-TC model was significantly better than those of the other models in both the internal and external datasets (*P *< .05). The MDT-TC achieved an AUC of 0.951 (95% CI = 0.943 to 0.960) on the internal test set, an AUC of 0.837 (95% CI = 0.779 to 0.894) on the FY set, an AUC of 0.816 (95% CI = 0.741 to 0.892) on the DZ set, and an AUC of 0.871 (95% CI = 0.829 to 0.912) on the SX set (Figure 3, A-D).

**Table 2.** The diagnostic performance of MDT-TC system on internal test set (SW, *n* = 2163) and 3 external test sets (DZ, *n* = 156; SX, *n* = 356; FY, *n* = 201).

Dataset	Method	Tn	Tp	Fn	Fp	Sensitivity	Specificity	Accuracy
SW (internal test set, *n* = 2163)	MMOE	274	1630	109	150	0.9373	0.6462	0.8803
	MMOE+Lightgbm	308	1711	28	116	0.9839	0.7264	0.9334
	MMOE+GBDT	263	1723	16	161	0.9908	0.6203	0.9182
	MMOE+XGBoost	261	1721	18	163	0.9896	0.6156	0.9163
	MDT-TC	278	1716	23	146	0.9868	0.6557	0.9219
DZ (external test set, *n* = 156)	MMOE	65	44	13	34	0.7719	0.6566	0.6987
	MMOE+Lightgbm	77	47	10	22	0.8247	0.7778	0.7949
	MMOE+GBDT	68	51	6	31	0.8947	0.6869	0.7628
	MMOE+XGBoost	71	48	9	28	0.8421	0.7172	0.7628
	MDT-TC	69	52	5	30	0.9122	0.6970	0.7756
SX (external test set, *n* = 356)	MMOE	31	247	63	15	0.7968	0.6739	0.7809
	MMOE+Lightgbm	35	261	49	11	0.8419	0.7609	0.8315
	MMOE+GBDT	29	293	17	17	0.9452	0.6304	0.9045
	MMOE+XGBoost	28	293	17	18	0.9452	0.6086	0.9017
	MDT-TC	27	280	30	19	0.9032	0.5870	0.9157
FY (external test set, *n* = 201)	MMOE	16	128	32	25	0.8000	0.3902	0.7164
	MMOE+Lightgbm	31	126	34	10	0.7875	0.7561	0.7811
	MMOE+GBDT	13	130	10	34	0.9286	0.2766	0.7647
	MMOE+XGBoost	15	136	4	32	0.9714	0.3191	0.8075
	MDT-TC	24	137	23	17	0.8563	0.5854	0.8010

Abbreviations: MMOE= Multi-gate Mixture-of-Experts; GBDT= Gradient Boosting Decision Tree; XGBoost= eXtreme Gradient Boosting; SW=Southwest; DZ= Dazu; SX=Shanxi; FY=Fuyang

**Figure 4.**
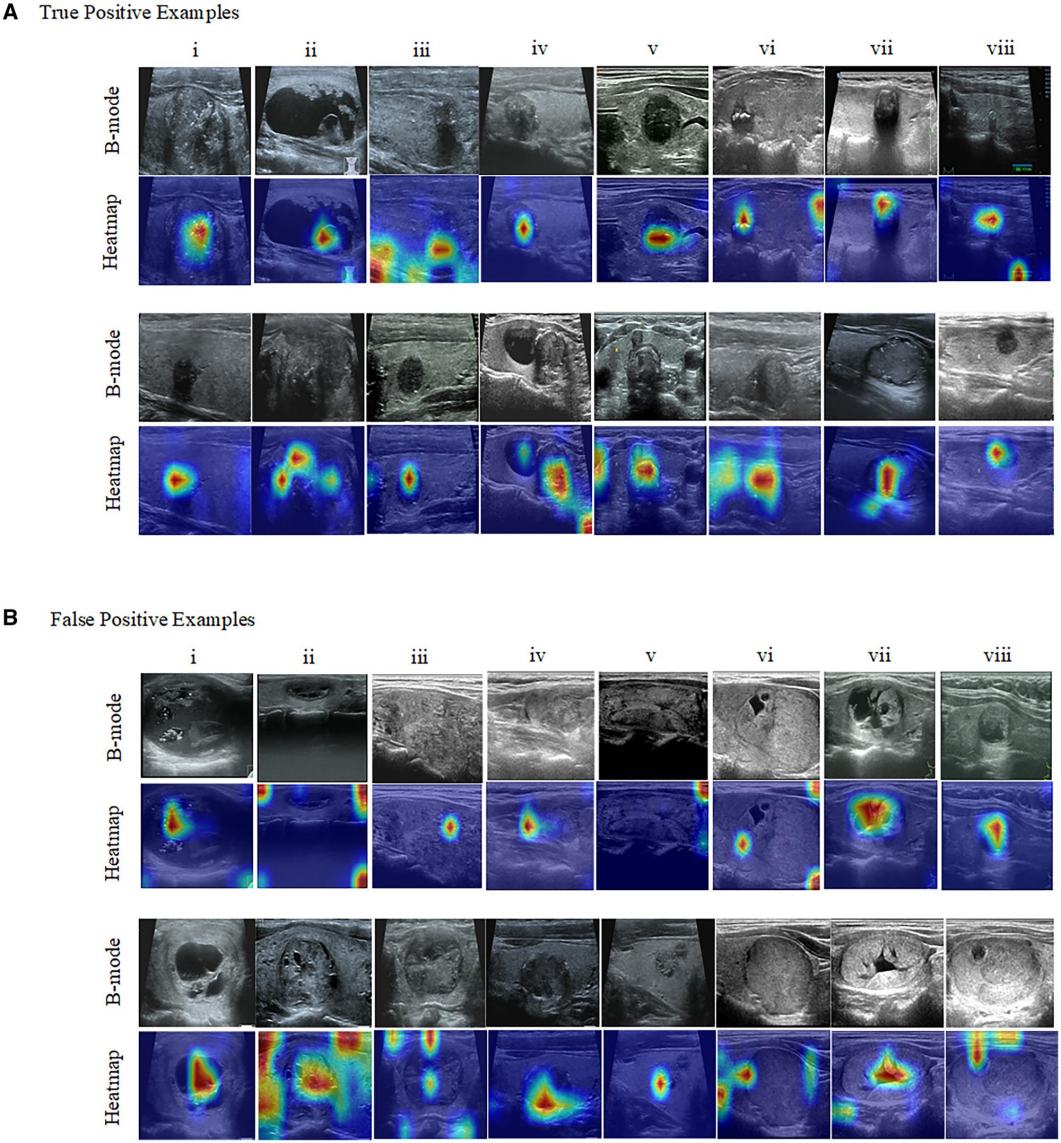
Visualization of the thyroid lesions attention heatmaps in B-mode ultrasound (US) images from the Southwest (SW) cohort. These heatmaps are generated using the deep convolution in MMOE. **A)** Heatmaps of true positive thyroid lesions, confirmed as malignant by both MDT-TC and pathology; **B)** heatmaps of false positive thyroid lesions, in which MDT-TC predicted malignant, while pathological results were benign.

Furthermore, the radar map depicts the balance of the model performance using the 5 diagnostic indices as the dimensions. The radar maps were constructed based on standard classification indicators, and binary classification indicated that the MMOE+Avg models had larger areas under the MDT-TC framework in the internal test set and 3 external test sets (Figure 3, E-H). These data suggest that the MDT-TC framework could be significantly better than other MMOE-based task modes. We further visualized the gradient heatmap in the deep convolution of MMOE. We found instances of true-positive and false-positive outcomes within the SW dataset (Figure 4). Our heat maps highlight areas in the US images that play a crucial role in distinguishing between malignant and benign tissues (red denotes high significance and blue denotes low significance). These visual aids offer an intuitive understanding of the learning process of the model from the training data by emphasizing the lesion regions. The numbers of false positives in the internal dataset and the 3 external datasets were 146, 17, 30, and 19, respectively (Table 2). In total, 8 false-positive cases showed strong signals (Figure 4, B and Figure S5).

### US features critical for decision making

We further evaluated 8 US features for decision making based on 3 decision tree models in the proposed MDT-TC framework. In the GBDT and XGBoost models, we found that the SHA features of thyroid nodules were the most important, reaching 0.69 and 0.56, respectively. In the LightGBM model, ECH was the most important factor, reaching 0.82. For the 3 decision tree models, MAC and PCL appear to have little effect on decision making. We also evaluated the learning effect of each feature in the first stage of the MDT-TC based on indicators such as accuracy and specificity (Figure S2 and Table S4). For features with higher importance, we determined that the model performs well. Specifically, the model achieved accuracy in determining echogenicity (ECH) attributes of up to 87.56% and 69.21% in identifying shape categories.

### Multifactorial exploration of AI assistance in practice

After conducting reliability analyses on multiple validation datasets (SW, SX, DZ, FY), we plotted the calibration curves for each validation dataset under MDT-TC (Figure 3, I-L). The specific results show that the calibration curves of the MDT-TC model in both the internal and external validation datasets are very close to the perfect calibration curve, demonstrating excellent performance. For the internal validation dataset (SW), the model’s Brier score and ECE were 0.101 and 0.069, respectively, indicating that the predicted probabilities of the model matched the actual results well. On the external validation dataset, the model’s calibration performance remained excellent, especially in the SX dataset, where the Brier score and ECE were 0.078 and 0.107, respectively, showing the most outstanding performance. In the calibration plot corrected for Bootstrap bias, we observed that the calibrated curve after bias correction showed a significant improvement compared with the original calibration curve. In certain intervals, the bias-corrected curve approached the diagonal, indicating more precise calibration results. We also further verified the stability and reliability of the model’s prediction results by plotting the confidence intervals for each probability interval.

To evaluate the clinical utility of the MDT-TC model, we developed an online collaborative diagnostic platform (Figure S6). Six radiologists with varying clinical experience (stratified into junior practitioners [<5 years] and senior specialists [>15 years]) participated in blinded diagnostic evaluations. Each radiologist performed comparative diagnostic assessments with and without assistance from the MDT-TC. The validation cohort comprised randomly selected imaging studies from 4 centers (SW, DZ, FY, and SX). To optimize workflow efficiency while maintaining diagnostic rigor, our evaluation protocol standardized the caseloads to approximately 200 anonymized studies per radiologist.

Interestingly, differences in accuracy between the 6 doctors before and after AI assistance were normally distributed (Shapiro-Wilk test, W = 0.956, *P* = .794). The paired-sample *t*-test indicated that the detection effect after using AI was significantly better than that without AI (*t* = −2.743, *P* < .05). With the exception of 1 doctor whose detection effect did not change significantly with the assistance of AI, the other 5 doctors achieved varying degrees of accuracy improvement. On average, the accuracy, sensitivity, PPV, NPV, F1, and Kappa of the 6 doctors increased from 0.762, 0.760, 0.908, 0.516, 0.82, and 0.448 to 0.783, 0.786, 0.913, 0.555, 0.838, and 0.505, respectively (Table 3). The clinical diagnostic ability was significantly improved with the assistance of the model.

**Table 3.** Comparison of diagnostic performance of the MDT-TC model, radiologists, and radiologists with the aid of the model.

	AUROC (95%CI)	*P*	Accuracy	Specificity	Sensitivity	PPV	NPV	Kappa	F1
Junior	0.726 (0.686 to 0.769)	.010	0.730 (0.691 to 0.765)	0.721	0.732 (0.691 to 0.771)	0.890	0.467	0.383	0.803
Junior with MDT-TC	0.753 (0.712 to 0.794)		0.756 (0.721 to 0.793)	0.748	0.759 (0.718 to 0.800)	0.903	0.502	0.435	0.825
Senior	0.800 (0.763 to 0.836)	.100	0.795 (0.761 to 0.825)	0.812	0.789 (0.751 to 0.829)	0.929	0.552	0.518	0.853
Senior with MDT-TC	0.807 (0.769 to 0.843)		0.811 (0.781 to 0.840)	0.799	0.816 (0.779 to 0.848)	0.927	0.868	0.545	0.868
Radiologists	0.763 (0.735 to 0.791)	.001	0.762 (0.738 to 0.785)	0.766	0.761 (0.735 to 0.788)	0.910	0.508	0.449	0.829
Radiologists with MDT-TC	0.780 (0.753 to 0.806)		0.784 (0.760 to 0.807)	0.773	0.787 (0.761 to 0.813)	0.915	0.540	0.489	0.846

Abbreviations: CI=Confidence Interval; AUROC= Area Under the Receiver Operating Characteristic curve; PPV=Positive Predictive Value; NPV=Negative Predictive Value.  Values in parentheses are 95% confidence intervals.

## Discussion

In this study, we designed an MDT-TC system to discriminate between benign and malignant thyroid nodules on the basis of US images and features. The experimental results indicated that, compared with the model using thyroid lesion B-mode US images combined with Doppler images, our MDT-TC system with B-mode US images of thyroid lesions could enhance radiologists’ accuracy and sensitivity, with an AUC of 0.903 (95% CI = 0.890 to 0.916). Previous studies suggest no significant difference in blood flow patterns between benign and malignant nodules using color Doppler and that flow detected by conventional Doppler US within nodules is insufficient for distinguishing benign from malignant lesions.^25^ This is likely because malignant nodules exhibit higher metabolic activity, characterized by numerous irregular, tortuous microvessels penetrating both the central and peripheral regions. Furthermore, neovascular vessels are slow-flow, microstructured vascular structures that are very small in size. Doppler US images measure the rapid flow of large and medium vessels and use a wall filter to prevent unnecessary signals.^26^ Doppler US methods consider the slow flow of microvascular vessels as an artifact and its filter detects signals from slow flow or microvessels as artifacts as well. These detections were then deleted.^27^^,^^28^ Therefore, using the MDT-TC system, we found that MMOE yielded the best diagnostic AUC performance when B-mode thyroid lesion US images were used.

The current guidelines recommend US as the preferred imaging method for thyroid nodule diagnosis. However, signs of benign and malignant thyroid nodules on US images often overlap.^29^ Most existing models give the user a static output—malignant vs benign—and are purely radiologically driven.^30^^,^^31^ However, accurate recognition and consistent interpretation of US features are challenging for less-experienced operators, resulting in moderate to substantial inter- and intra-observer variability. The malignancy risk estimated by US is not determined by a single US predictor but by a combination of coexisting thyroid nodule US features.^32^

Importantly, COM, ECH, SHA, MAR, and MAC were all associated with TC diagnoses, and these features are important for traditional risk stratification. In our AI diagnostic system, the importance of these US features varies in the 3 decision tree models’ MDT-TC framework, with ECH and SHA likely to be the more important features. Our data were similar to previous research that suggests that US predictors for malignancy are dependent on thyroid nodule ECH and SHA. Specifically, suspicious US features, including taller-than-wide lesions and solid hypoechoic nodules, are strongly associated with an increased risk of malignancy.^32-34^ Ultrasound image interpretation is subject to substantial interobserver variability, particularly among physicians with limited US experience or non-imaging specialists. Our MDT-TC system not only outputs benign and malignant probabilities for thyroid lesions but also provides critical US features for radiologists to reference. Therefore, our system has the potential to mitigate operator dependency in thyroid nodule image interpretation, thereby supporting more objective and accurate malignancy risk assessment and facilitating evidence-informed FNA decision making.

We comprehensively evaluated the calibration performance of the MDT-TC model through reliability plots and Bootstrap bias correction calibration plots. The calibration analysis revealed that the MDT-TC model exhibited stable and reliable performance across different datasets and could effectively assist doctors in making judgments in clinical practice. Our study revealed that the MDT-TC system yielded satisfactory performance in thyroid nodule diagnosis, with an AUC of 0.951, accuracy of 92.6%, and sensitivity of 98.7% for the validation data set from the internal data test. We also found the following: an AUC of 0.837, accuracy of 80.1%, and sensitivity of 85.6% for the external test data set from the FY hospital; an AUC of 0.816, accuracy of 77.6%, and sensitivity of 91.2% for the external test data set from the DZ hospital; and an AUC of 0.871, an accuracy of 91.6%, and a sensitivity of 90.3% for the external test data set from the SX hospital. These results suggest that our MDT-TC system can improve diagnostic accuracy.

The higher performance we saw in the MDT-TC system in this study may be attributed to its MMOE framework, which splits the underlying shared network layers into multiple shared expert networks and learns their contributions to different tasks.^22^ Because of the flexible sharing of parameters in multitask learning, the accuracy and stability of the MMoE model are likely better than the single task models when the nodes of their networks are similar.^35^ Our study provided evidence that the multi-expert networks mixing and information sharing mechanism within the MMoE model can capture prediction task differences and improve prediction accuracy. We found that deploying the MMoE model on thyroid nodule identification platforms can effectively improve thyroid nodule diagnostic accuracy and facilitate its application in clinical diagnosis and treatment.

The assistance of MDT-TC in our study increased the radiologist’s accuracy from 0.762 to 0.784, and the sensitivity of the 6 radiologists increased from 0.766 to 0.773 (Table 3). MDT-TC also improved the performance of radiologists in clinical practice. The diagnostic accuracy of the US radiologists when assessing thyroid lesions assisted by the MDT-TC system was significantly higher than that of the non-assisted MDT-TC system (*P *< .001) (Table 3). Stratified analysis was conducted based on the doctors’ clinical experiences, and we found that the diagnostic accuracy of thyroid lesions in junior doctors via the MDT-TC system was significantly higher than that of junior doctors alone (*P *< .01) (Table 3). However, senior doctors showed no significant difference in thyroid lesion diagnostic accuracy when using MTD-TC (*P *= .10) (Table 3). Therefore, the use of the MDT-TC system for thyroid lesion screening and detection is of great significance for junior doctors. The MDT-TC system we proposed can be used to evaluate thyroid lesions, and its performance is comparable to that of experienced human experts. By providing US image features and the malignancy probability of lesions generated by the model, the diagnostic accuracy of junior physicians in identifying thyroid lesions can be enhanced. Although the absolute improvement in performance metrics for junior radiologists was modest, the consistent and statistically significant enhancement underscores the potential clinical utility of MDT-TC as a supportive tool in their diagnostic workflow. Therefore, the MDT-TC system could contribute to discriminating against the high risk of thyroid lesions and reduce misdiagnosis. Notably, the most crucial role of the proposed model is to improve diagnostic accuracy by assisting clinicians. Despite its reported performance, MDT-TC does not supplant the essential role of clinicians, who retain ultimate authority in clinical decision making. In cases of disagreement, the final diagnosis was determined by a senior radiologist who reviewed all available information independently.

However, this study has some limitations. First, its retrospective design introduced differences in class distributions across datasets as it combined consecutive patient series and convenience samples. Second, there was an inevitable selection bias, as we only included thyroid nodules that were definitively classified as benign or malignant based on biopsy cytology or surgical pathology results. The accuracy and stability of AI models depend on the quality of cytopathological diagnoses. The majority of benign nodules that did not undergo US-guided FNA biopsy was excluded. In our clinical practice, such nodules are typically recommended for US follow-up. Third, our model’s training process incorporated only the patients’ thyroid nodule US images and did not include other clinical data related to diagnosis and treatment. Moreover, the relatively modest sample sizes of the 3 external validation sets, combined with the heterogeneity in US equipment and operator expertise, may not adequately account for TC incidence rates across different regional populations. These factors could compromise the model’s predictive accuracy for TC or reduce the generalizability of the results. Finally, there are potential biases in the selection of radiologists and patient data, including the exclusion of low-quality images and normal scans. Consequently, the validation of future prospective large-scale clinical screening cohorts is necessary.

In conclusion, the MDT-TC system significantly improves the diagnostic accuracy of thyroid nodule differentiation. The proposed model can extract US features from thyroid nodules, conduct effective and objective image analysis, and provide US features and precise outcome predictions for TCs. The performance of this model in these clinical cases shows its effectiveness in assisting physicians in practice, especially junior doctors, who are training for more accurate diagnostic capabilities.

## Supplementary Material

pkag037_Supplementary_Data

## Data Availability

All codes used for model development and training are available at: https://github.com/Big-Fish-And-Small-Shrimp/tirads. A demonstration showing AI-assisted diagnosis based on MDT-TC is available at https://huggingface.co/spaces/ruixuezhang/SWThyroidAISystem. The trained model is available for review upon request. All datasets in the current study were used with licenses from the respective hospital systems and were not publicly available.
