# Supplementary material for: Artificial intelligence-based multimodal multitask analysis of thyroid ultrasound image features predicts thyroid cancer: a multicenter study
Source: JNCI Cancer Spectr. 2026 Apr 9;10(4):pkag037. doi: 10.1093/jncics/pkag037 (PMC13341012; doi:10.1093/jncics/pkag037)
Supplement: pkag037_Supplementary_Data [file pkag037_supplementary_data.docx]

**Supplementary Materials**

#### Additional details on data preprocessing

To enhance the dataset for training the deep learning model, we implemented a comprehensive image preprocessing pipeline. Initially, all images were resized to a uniform resolution of 270×190 pixels using the Lanczos interpolation algorithm to preserve structural details. To mitigate overfitting and improve model generalization, we applied randomized data augmentation techniques. These included brightness adjustment (with a scaling factor sampled from [0.8, 1.2]), contrast enhancement (factor range [0.7, 1.3]), and saturation modification (factor range [0.6, 1.4]). Additionally, we introduced non-linear color transformations through hue shifting (±10% of the full spectrum) and adaptive white balance correction based on predefined blackbody chromaticity coordinates (6500K ± 2000K). For geometric augmentation, we integrated image rotation (±15°) and shearing (±10% distortion) by implicitly modifying pixel coordinates during the adjustment process. All transformations were dynamically applied during training using a probabilistic framework to ensure diversity in augmented samples while maintaining anatomical plausibility. Pixel values were normalized to the [0, 1] range post-augmentation to standardize input distributions.

#### model construction and procedures

The proposed model architecture adopts a Multi-gate Mixture-of-Experts Tree-based Model Career Classification(MDT-TC) framework with dual ResNet50 backbones pretrained on ImageNet. Each backbone processes B-mode and color Doppler ultrasound images independently, generating 2048-dimensional feature vectors that are concatenated into a unified 4096-dimensional representation. To enhance robustness, we implemented a Multi-gate Mixture-of-Experts (MMOE) structure containing 9 expert networks and task-specific gating mechanisms, enabling simultaneous optimization of nine clinical classification tasks (3-5 classes per task). The final classification layer consists of two fully connected layers (800 to [2-5] neurons depending on task) with ReLU activation and a 0.3 dropout ratio after the first FC layer to prevent overfitting.

To address the severe class imbalance (5704 malignant vs. 1500 benign samples), we developed an Ensemble Deep Learning with Bagging Compensation (EDL-BC) framework. This involved: 1) Creating 30 balanced bootstrap subsets (5704 malignant + 1500 benign samples each); 2) Training independent MSFLM instances on each subset; 3) Aggregating predictions through soft voting:

$$H(x)=\frac{1}{30}\sum_{i=1}^{30} h_{i}^{'}(x)$$

where $h_{i}^{'}(x)$ represents the probabilistic output from the i-th learner. The model was optimized using Adam with an initial learning rate of 0.0001 (weight decay=0.0004) over 100 epochs (batch size=36), incorporating automatic mixed precision training for computational efficiency. For critical malignancy prediction (Task 8), we further enhanced performance through gradient-boosting ensemble integration - combining MMOE outputs with XGBoost (η=0.1, max_depth=8), LightGBM (num_leaves=30), and GBDT (n_estimators=70) predictions using a weighted fusion strategy (30% DL + 70% tree-based probabilities). Model validation employed 5-fold cross-validation with comprehensive metrics including AUC-ROC (with 95% confidence intervals calculated via DeLong's method), sensitivity, and specificity.

#### Calibration Analysis

We used the Brier score to measure the overall difference between the predicted probabilities and the actual outcomes, and the Expected Calibration Error (ECE) to quantify the average discrepancy between predicted and empirical probabilities.

$$Brier=\frac{1}{N}\sum_{i=1}^{N} {(y_{i}-p_{i})}^{2}$$

Where N is the total number of samples, $y_{i}$ is the actual label (0 or 1) for the i-th sample, and $p_{i}$ is the predicted malignancy probability for the i-th sample.

$$ECE=\sum_{k=1}^{M} \frac{\left| S_{k} \right|}{N}\left| \bar{p}_{k}-\frac{1}{\left| S_{k} \right|}\sum_{i\in S_{k}} y_{i} \right|$$

Where N is the total number of samples, M is the number of bins (here, M=10), $S_{k}$ is the set of samples in the k-th bin, $\left| S_{k} \right|$ is the number of samples in the k-th bin, $\bar{p}_{k}$ is the average predicted probability within the k-th bin, and $\frac{1}{\left| S_{k} \right|}\sum_{i\in S_{k}} y_{i}$ is the actual observed malignancy proportion within the k-th bin.

To further enhance the robustness of the calibration assessment, we generated Bootstrap bias-corrected calibration plots. This method involves calculating the observed values for each probability bin, performing Bootstrap resampling, and deriving a bias-corrected calibration curve. It allows for the estimation of confidence intervals for the calibration plot, thereby improving the reliability of the evaluation. Specifically, the predicted probabilities were binned, the average predicted probability and actual malignancy rate were computed for each bin, and the Bootstrap method was applied to generate multiple samples. Calibration curves were calculated for each Bootstrap sample to obtain the average curve and its confidence intervals across probability bins.

#### Model interpretability

To enhance the model interpretability and visualize decision-making patterns, we implemented Gradient-weighted Class Activation Mapping (Grad-CAM) to generate attention heatmaps for critical lesion regions. For a target class c, we first captured feature maps $A^{k}$ from the penultimate convolutional layer (specifically the 6th layer in the VGG11 architecture) through forward propagation hooks. Concurrently, backward gradients $\frac{\partial y^{c}}{\partial A^{k}}$ were recorded using gradient hooks, where $y^{c}$ denotes the model's prediction score for class c. The channel-wise importance weights $A^{k}$ were computed via global average pooling of gradients:

$$a_{k}^{c}=\frac{1}{Z}\sum_{i,j} \frac{\partial y^{c}}{\partial A_{i,j}^{k}},$$

where Z represents the spatial dimensions of the feature maps. The preliminary heatmap was then generated through a linear combination of feature maps and weights, followed by ReLU rectification to emphasize positive influences:

$$h_{Grad-CAM}^{c}=ReLU(\sum_{k} a_{k}^{c}A^{k})$$

The raw heatmap was resized to match the input resolution (270×190 pixels) using bicubic interpolation and normalized to [0, 1] for visualization. To fuse semantic and spatial information, the normalized heatmap was superimposed onto the original ultrasound image via alpha blending (weight=0.5) and color-coded using a jet colormap. This pipeline enabled intuitive identification of diagnostically relevant regions, such as malignant lesion boundaries and vascular patterns, aligning with clinical annotations. The implementation leveraged PyTorch hook mechanisms to dynamically capture intermediate activations and gradients during inference, ensuring computational efficiency without architectural modification. Validation on test cases confirmed that the highlighted regions consistently correlated with pathologically confirmed lesion characteristics, demonstrating the model's clinically interpretable feature localization capability.

**Supplementary Figures and Tables Legends**

**Supplementary Figure S1.** Flow chart of patient selection.

**Supplementary Figure S2.** Comparison of conventional and MDT-TC auxiliary diagnosis. Our model included eight clinical ultrasound (US) images, including composition (COM), echogenicity (ECH), shape (SHA), margin (MAR), echogenic foci or large comet-tail artifact (ELCA), macrocalcification (MAC), peripheral (rim) calcifications (PCL), and punctate echogenic foci (PEF).

**Supplementary Figure S3.** Comparison between the models. We conducted comparison experiments between the traditional single-task image classification model (i.e., EfficientNet, GoogleNet, ResNet18, ResNet50, SE_ ResNet18, SE_ ResNet50, VGG11, and VGG16) and the MMOE architecture with multi-task learning model. During this process, we leveraged the average area under the receiver operating characteristic curve (AUC) as the primary metric. We observe that MMOE achieved the optimal result of the average AUC. Therefore, we employed that model in the proposed Multi-head Model in our MDT-TC system.

**Supplementary Figure S4.** Experimental results with various inputs. We fed B-mode ultrasound (US) images, Color Doppler US images, and B-mode US images + Color Doppler US images into the Multi-gate Mixture-of-Experts (MMOE). We found that MMOE yielded the best outcome of AUC when using B-mode US images of thyroid lesions. Multi-source feature learning and single-source feature learning were statistically different (P <0.05).

**Supplementary Figure S5.** Attention heatmaps of images that were predicted as (true/false) negative lesions by MMOE. (a) Heatmaps of true negative thyroid lesions, in which both the MDT-TC system and pathology confirmed the lesions as negative; (b) heatmaps of false negative thyroid lesions, in which MDT-TC predicted as negative while pathological results were malignant.

**Supplementary Figure S6.** Schematic diagram of AI-assisted diagnosis of thyroid gland based on MDT-TC.

**Supplementary Table S1.** Summary of ultrasound (US) devices. The US images in the SW dataset and the three external datasets were scanned using different Doppler US devices. There was no bias in terms of device preference for the training and validation datasets in this study.

**Supplementary Table S2.** Performance of different backbone networks cross-validated on datasets for predicting malignant thyroid lesions on the southwest (SW) dataset.

**Supplementary Table S3.** Performance of single- and multi-source inputs in MMOE for thyroid lesion malignancy prediction using 5-fold cross-validation on the southwest (SW) dataset.

**Supplementary Table S4.** Diagnostic performance of the MDT-TC system for each clinical ultrasound imaging feature.

Table S1. Summary of the ultrasound (US) devices. The US images in the SW dataset and three external datasets were scanned by different Doppler US devices. There was no bias in terms of device preference for the training and validation datasets in this study.

| Hosptial | Equipment | Numbers |
| --- | --- | --- |
| SW | GE E9 (General Electric Co., USA) | 1671 |
| SW | Siemens (Siemens Healthcare GmbH, USA) | 3022 |
| SW | Philips EPIQ7 (Philips Medical Systems, Andover, MA) | 1592 |
| SW | Mindray R9 Mindray Resona 9 (Mindray, Shenzhen, China) | 599 |
| DZ | GE E9, (General Electric Co., USA) | 156 |
| SX | Philips EPIQ7, (Philips Medical Systems, Andover, MA) | 346 |
| FY | Philips EPIQ7 (Philips Medical Systems, Andover, MA) | 201 |

Table S2. Performance of different backbone networks cross-validated on data sets for predicting malignant thyroid lesions on the Southwest (SW) dataset.

| Method | Sensitivity（%） | Specificity（%） | Accuracy（%） | AUC |
| --- | --- | --- | --- | --- |
| VGG11 | 0.9086 | 0.6792 | 0.8636 | 0.890(0.877~0.904) |
| VGG16 | 0.9551 | 0.5849 | 0.8826 | 0.883(0.868~0.897) |
| EfficientNet | 0.9137 | 0.5189 | 0.8363 | 0.822(0.803~0.841) |
| GoogleNet | 0.9017 | 0.6580 | 0.8539 | 0.869(0.854~0.884) |
| ResNet18 | 0.9390 | 0.4741 | 0.8479 | 0.846(0.829~0.863) |
| ResNet50 | 0.8965 | 0.5495 | 0.8285 | 0.821(0.803~0.840) |
| SE_ResNet18 | 0.8832 | 0.6085 | 0.8294 | 0.835(0.817~0.852) |
| SE_ResNet50 | 0.9103 | 0.4646 | 0.8229 | 0.813(0.794~0.833) |
| **MMOE** | **0.9258** | **0.6981** | **0.8812** | **0.903(0.890~0.916)** |

Table S3. Performance of single-source and multi-source inputs in MMOE for malignancy prediction of thyroid lesion using 5-fold cross-validation on the Southwest (SW) dataset.

| Model | Tn | Tp | Fn | Fp | Sensitivity | Specificity | Accuracy |
| --- | --- | --- | --- | --- | --- | --- | --- |
| Single B-mode MMOE | 274 | 1630 | 109 | 150 | 0.9373 | 0.6462 | 0.8803 |
| Single color Doppler MMOE | 269 | 1300 | 439 | 155 | 0.7476 | 0.6344 | 0.7254 |
| Multimodal MMOE | 266 | 1520 | 219 | 158 | 0.8741 | 0.6274 | 0.8257 |

Table S4. The diagnostic performance of MDT-TC system on each clinical ultrasound images indicators.

| Other indicators | Tn | Tp | Fn | Fp | Sensitivity（%） | Specificity（%） | Accuracy（%） |
| --- | --- | --- | --- | --- | --- | --- | --- |
| COM | 167 | 1392 | 205 | 399 | 0.8716 | 0.2951 | 0.7208 |
| ECH | 75 | 1819 | 66 | 203 | 0.9650 | 0.2698 | 0.8756 |
| SHA | 1264 | 233 | 391 | 275 | 0.3734 | 0.8213 | 0.6921 |
| MAC | 1457 | 6 | 156 | 544 | 0.0370 | 0.7281 | 0.6764 |
| ELCA | 1505 | 111 | 424 | 123 | 0.2075 | 0.9244 | 0.7471 |
| MAC | 2160 | 0 | 3 | 0 | 0.0000 | 1.0000 | 0.9986 |
| PCL | 2160 | 0 | 3 | 0 | 0.0000 | 1.0000 | 0.9986 |
| PEF | 511 | 750 | 392 | 510 | 0.6567 | 0.5005 | 0.5830 |


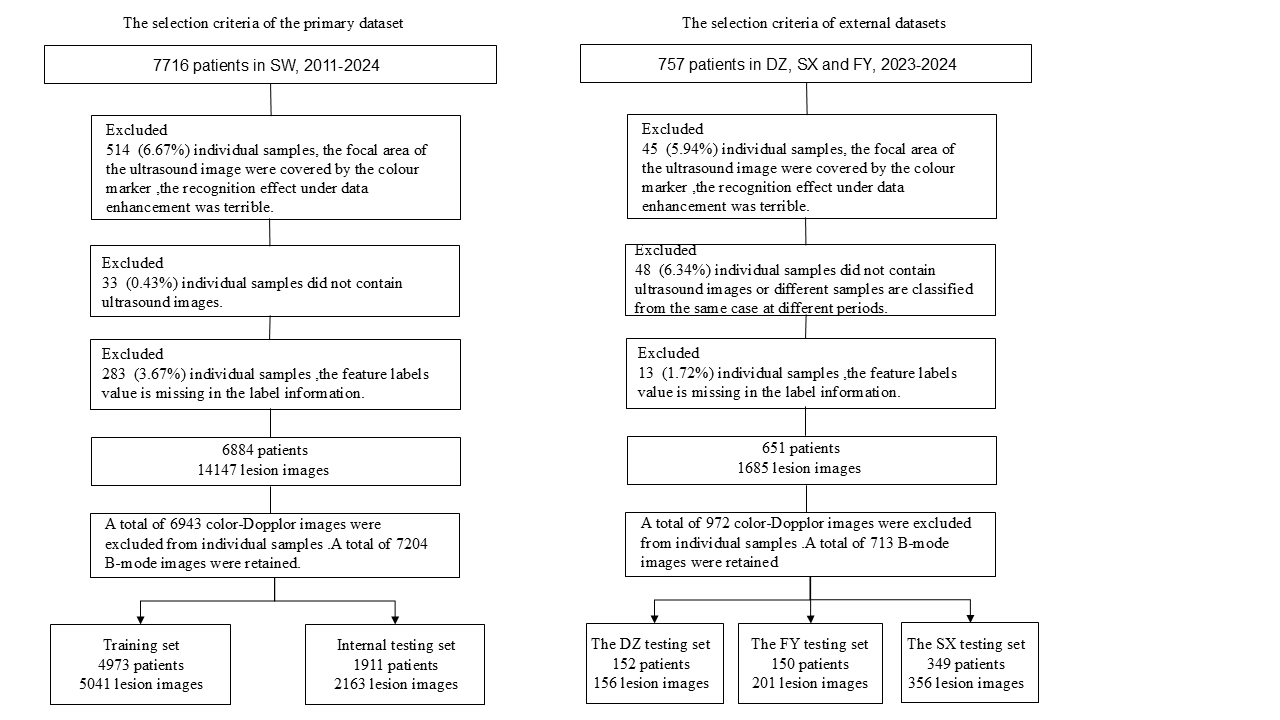


Figure S1. Flow chart of patients’ selection.


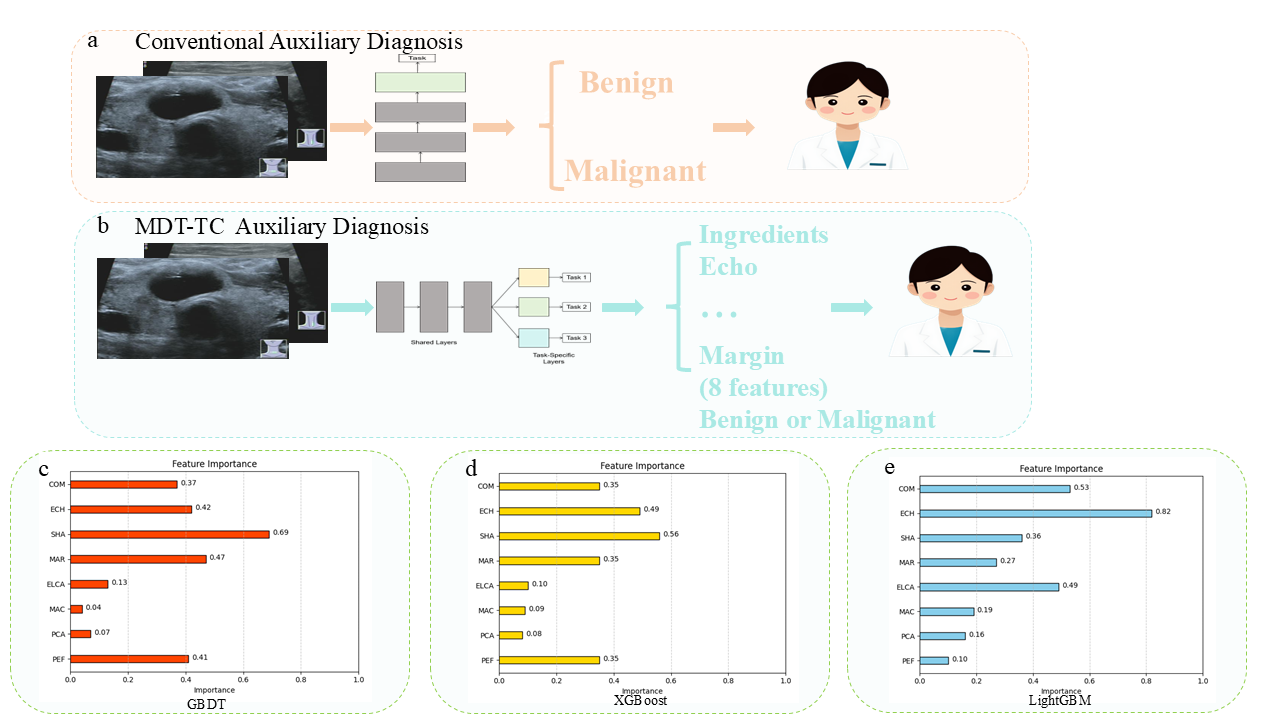


Figure S2. The comparison of conventional and MDT-TC auxiliary diagnosis. Our model included eight clinical ultrasound images features such as composition (COM), echogenicity (ECH), shape (SHA), margin (MAR), echogenic foci or large comet-tail artifact (ELCA), macrocalcification (MAC), peripheral (rim) calcifications (PCL), and punctate echogenic foci (PEF).


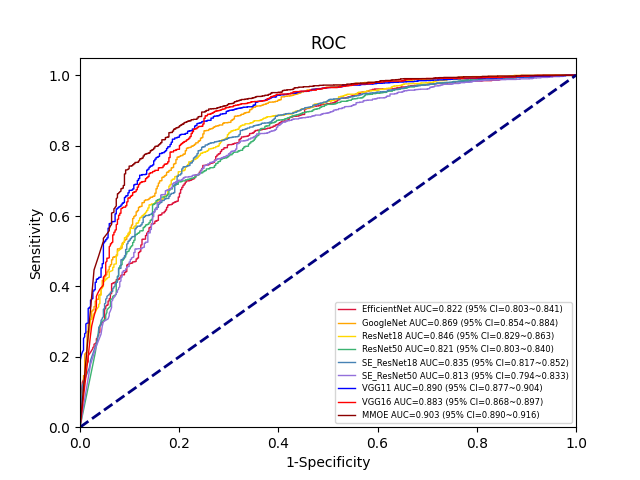


Figure S3. Comparison between the models. We conducted the comparison experiments between the traditional single-task image classification model, namely EfficientNet, GoogleNet, ResNet18, ResNet50, SE_ ResNet18, SE_ ResNet50, VGG11 and VGG16, and the MMOE architecture with multi-task learning model. During this process, we leveraged the average area under the receiver operating characteristic curve (AUC) as the primary metric. We can observe that MMOE achieved the optimal result of average AUC. Therefore, we employed it in the proposed Multi-head Model in our MDT-TC system.


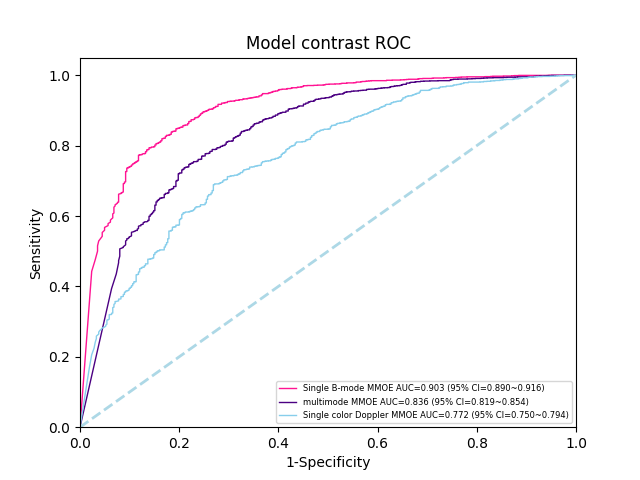
Figure S4. Experimental results with a variety of inputs. We fed B-mode US images, Color Doppler US images, and both of them into the Multi-gate Mixture-of-Experts (MMOE), respectively. It showed that the MMOE yielded the best outcome of AUC when using B-mode ultrasound (US) images of thyroid lesions. There was a significant statistical difference between multi-source feature learning and single-source feature learning (P <0.05).


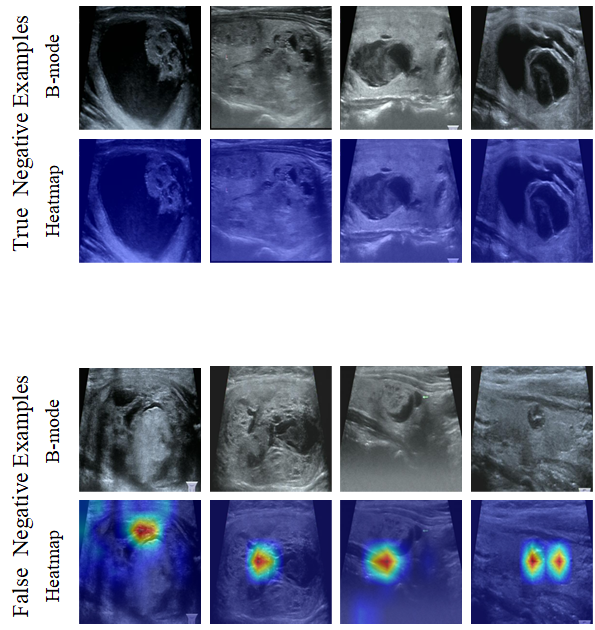


Figure S5. Attention heatmaps of images that were predicted as (true/false) negative lesions by MMOE. a. Heatmaps of true negative thyroid lesions. b. Heatmaps of false negative thyroid lesions.


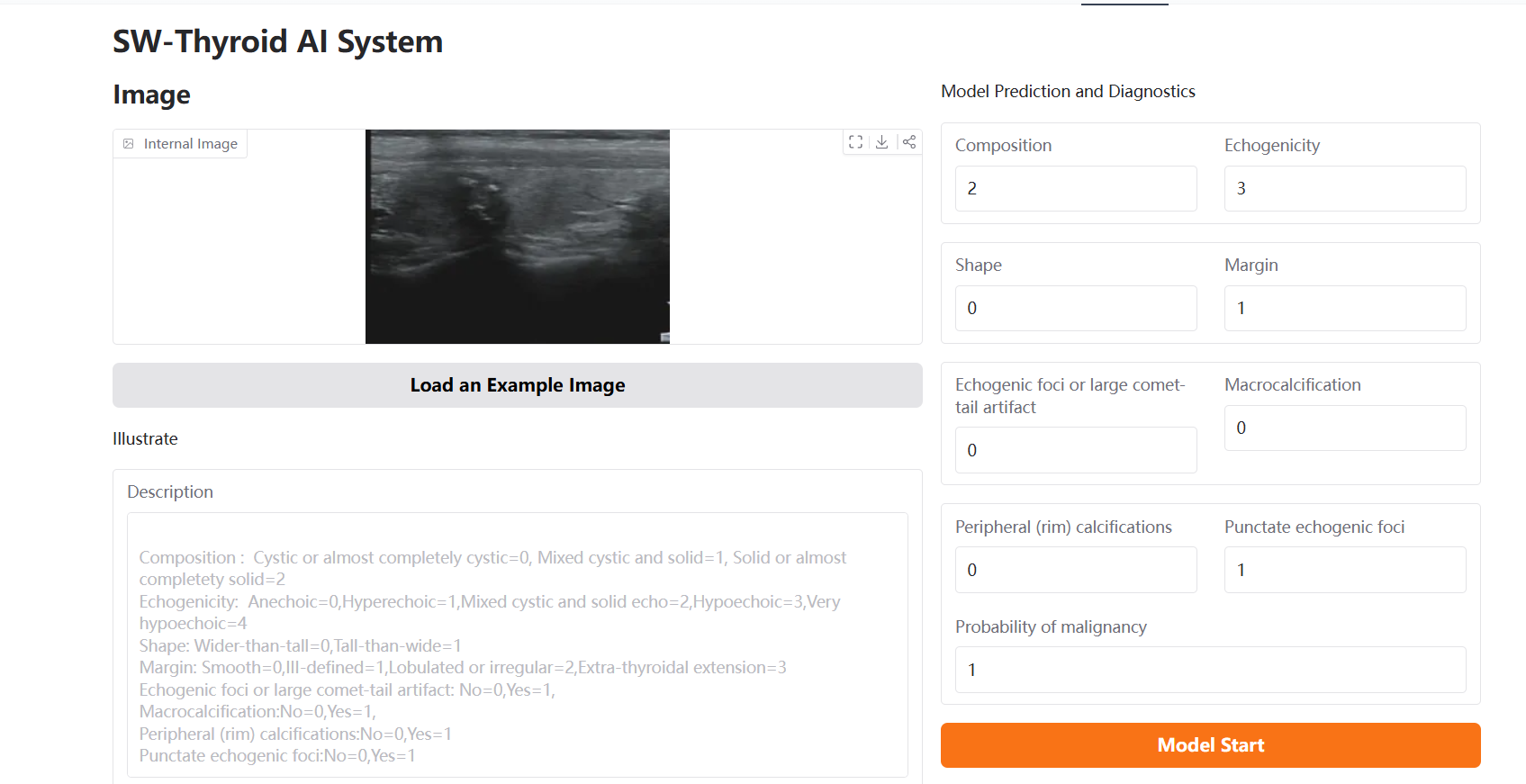


Figure S6. Schematic diagram of AI-assisted diagnosis of thyroid gland based on MDT-TC.
